# Human–Robot–Environment Interaction Interface for Smart Walker Assisted Gait: AGoRA Walker

**DOI:** 10.3390/s19132897

**Published:** 2019-06-30

**Authors:** Sergio D. Sierra M., Mario Garzón, Marcela Múnera, Carlos A. Cifuentes

**Affiliations:** 1 Department of Biomedical Engineering, Colombian School of Engineering Julio Garavito, Bogota 111166, Colombia; 2 INRIA, University Grenoble Alpes, Grenoble INP, 38000 Grenoble, France

**Keywords:** smart walker, human–robot–environment interaction, control strategies, shared control, gait assistance, gait rehabilitation

## Abstract

The constant growth of the population with mobility impairments has led to the development of several gait assistance devices. Among these, smart walkers have emerged to provide physical and cognitive interactions during rehabilitation and assistance therapies, by means of robotic and electronic technologies. In this sense, this paper presents the development and implementation of a human–robot–environment interface on a robotic platform that emulates a smart walker, the *AGoRA Walker*. The interface includes modules such as a navigation system, a human detection system, a safety rules system, a user interaction system, a social interaction system and a set of autonomous and shared control strategies. The interface was validated through several tests on healthy volunteers with no gait impairments. The platform performance and usability was assessed, finding natural and intuitive interaction over the implemented control strategies.

## 1. Introduction

Human mobility is a complex behavior that involves not only the musculoskeletal system but also dissociable neuronal systems. These systems control gait initiation, planning, and execution, while adapting them to satisfy motivational and environmental demands [1]. However, there are some health conditions and pathologies that affect key components of mobility [2] (e.g., gait balance, control, and stability [3]). Among these pathologies, Spinal Cord Injury (SCI), Cerebral Palsy (CP) and Stroke are found to be strongly related to locomotion impairments [4]. Likewise, the progressive deterioration of cognitive functions [1] (i.e., sensory deficits and coordination difficulties [5]) and the neuromuscular system in the elderly [6] (i.e., loss of muscle strength and reduced effort capacity [5]) are commonly related to the partial or total loss of locomotion capacities.

Moreover, according to the World Health Organization (WHO) the proportion of the mobility impaired population has been experiencing constant and major growth [7]. Specifically, nearly 15% of the world’s population experience some form of disability [8], and by 2050 the proportion of the world’s population over 60 years will nearly double from 12% to 22% [9,10]. These studies also report that a larger percentage of this growth will take place in developing countries [9]. Although these populations may be represented by different types of disability, mobility impairments have been identified as a common condition in elderly populations and people with functioning and cognitive disabilities [5,11,12]. Considering this, several rehabilitation and assistance devices have been developed to retrain, empower or provide the affected or residual locomotion capacities [13].

Devices such as canes, crutches, walkers, and wheelchairs, as well as ambulatory training devices, are commonly found in assisted gait and rehabilitation scenarios [14] and are intended to improve user’s life quality. Concretely, mobility assistive devices are aimed at overcoming and compensating physical limitations by maintaining or improving individual’s functioning and independence in both clinical and everyday scenarios [15]. Regarding conventional walkers, these devices exhibit simple and affordable mechanical structures, as well as partial body weight support and stability. However, natural balance, user’s energetic costs, fall prevention and security issues are often compromised with conventional walkers [16]. Moreover, several issues related to sensory and cognitive assistance, often required by people with physical limitations, are not completely addressed by conventional devices [17,18,19]. Accordingly, to outstrip such problems, robotic technologies and electronics have been integrated, leading to the emergence of *intelligent walkers* or *Smart Walkers* (SWs).

The SWs are often equipped with actuators and sensory modalities that provide biomechanical monitoring mechanisms and individual’s intention estimators for user interaction, as well as several control strategies for movement and assistance level control [16]. Likewise, path following modules are usually included, in addition to safety rules and fall prevention systems [20]. These features enable SWs to interact in dynamic and complex environments. The particular selection and implementation of such features can be referred to as Human–Robot Interaction (HRI) interfaces [21]. Notwithstanding, Human–Robot–Environment Interaction (HREI) interfaces are required, in such a way that they provide natural user interactions, as well as effective environment sensing and adaption while maintaining safety requirements.

In this context, the design and implementation of a multimodal HREI interface for an SW is presented. Such implementation was made to improve previous implementations of HRI interfaces on SWs, by providing safety, natural user interactions and robust environment interactions. The HREI was focused on the development of shared control strategies (i.e., natural and intuitive user interaction while multiple systems are running), as well as on the implementation of a robust Robot–Environment Interaction (REI) interface (i.e., a safety system for collision prevention, a navigation system and a social interaction system). Moreover, the interaction interface was equipped with several strategies for therapy management and supervision by a technical or health care professional. To this end, several robotic and image processing techniques, as well as different control strategies, were implemented. Navigation and human detection systems were aimed at enabling the SW with social interaction and social acceptance capabilities. Additionally, user interaction systems and shared control strategies sought to provide a more natural, intuitive and comfortable interaction.

The remainder of this work is organized as follows. Section 2 describes the existing HRI and REI interfaces implemented on several SWs. Section 3 shows the proposed HREI interface and the platform description. Since the HREI interface is composed by a HRI interface and a REI interface, Section 4 describes the systems and modules for HRI on the *AGoRA Walker*, and Section 5 presents the systems for environment and social interaction (i.e., the REI interface). Thereafter, Section 6 details the different control strategies implemented on the HREI interface, while Section 7 exhibits the experimental test conducted to assess the interface performance. Finally, Section 8 expresses the conclusions and relevant findings of this work and mentions proposals for future research.

## 2. Related Work

Reviewing literature evidence, several SWs and walker based robotic platforms have introduced HRI and REI interfaces. Generally, these systems are aimed at assessing the user’s state (i.e., biomechanical and spatiotemporal parameters), the user’s intentions of movement and environment constraints. Likewise, these interfaces and interaction systems are commonly aimed at providing effectiveness, comfort, safety and different control strategies during rehabilitation and assistance tasks. For this purpose, some sensory modalities are frequently implemented, such as potentiometers, joysticks, force sensors, voice recognition modules and scanning sensors [20]. Some of these HRI and REI interfaces are shown in Table 1, where the SWs are characterized by their type (i.e, active for motorized walkers and passive for non motorized walkers), the sensors used, the internal modules (i.e., main reported functionalities or systems), the reported modes of operation, the implemented shared control strategies and by their social interaction capabilities (i.e., specific strategies for people avoidance or interaction).

One of the most notable smart walkers is CO-Operative Locomotion Aide (COOL Aide), which is a three-wheeled passive SW [36] intended to assist the elderly with routine walking tasks. It includes mapping and obstacle detection systems, as well as navigation and guidance algorithms. Additionally, it is equipped with force sensors on its handlebars and a Laser Range Finder (LRF) to estimate the user’s desired direction to turn. Although it is a passive walker, shared control strategies are achieved by granting walker control to the platform or the user.

Other passive walkers, such as those presented in [37,38], include navigation and guidance algorithms in conjunction with shared control systems. These strategies are based on sharing the steering control between the user and the walker.

Different approaches on active SWs have been developed in the past few years regarding HRI and REI interfaces [21,22,23,24,25,26,28,29,30,31,33]. These interfaces are also equipped with navigation and user interaction systems to provide shared control capabilities. Such strategies are based on granting walker steering to the user or the SW, depending on the obstacle detection and navigation systems, as well as on changing the walker responses to user’s commands (i.e., some strategies are based on inducing the user’s actions through haptic communication channels). To this end, user interaction systems are required to manage how user’s intentions of movement are interpreted. The estimation of such intentions is commonly achieved by admittance control systems, gait analysis systems, and rule-based algorithms.

In addition, other robotic walkers have been reported in the literature, including different HRI interfaces [41,42,43,44]. For instance, the approach developed by Ye et al. [42] includes a width changeable walker that adapts to the user’s intentions and environment constraints. Likewise, some REI interfaces have been presented in [45,46,47]. These approaches intend to assess the environment information to adapt their control strategies. Finally, regarding social interaction approaches, the *c-Walker* [40] includes a social force model that represents pedestrians and desired trajectory paths as repulsive or attractive objects, respectively. Although the *c-Walker* presents both shared control strategies and social interaction, it is a passive walker and its shared strategy is based on brakes control and shared steering of the platform.

According to the above, this work presents the implementation of an HREI interface in order to join the multiple advantages of the current HRI and REI interfaces on the AGoRA Smart Walker. The AGoRA Walker is equipped with a sensory and actuation interface that enables the implementation of several functionalities for HRI and REI, as well as a set of control strategies for shared control and social interaction. Moreover, the developed interface is equipped with a robust navigation system, a user interaction system (i.e., a gait analyzer module and an user’s intention detector), a low-level safety system, a people detection system for social interaction, and a safe strategy for shared control of the walker.

## 3. Human–Robot–Environment Interaction (HREI) Interface

### 3.1. Robotic Platform Description

According to the different motivations and related approaches presented in Section 1 and Section 2, this work covers the design, development, and implementation of a set of control strategies and interaction systems that establish an HREI interface on a robotic walker. Hence, a robotic platform was adapted to emulate the structural frame of a conventional assistance walker, by attaching two forearm support handlebars on the platform’s main deck. Specifically, the Pioneer LX research platform (Omron Adept Technologies, Pleasanton, CA, USA), named as *AGoRA Smart Walker*, was used to implement and test the interface systems. The platform is equipped with an onboard computer running a Linux operating system distribution providing support for the Robotic Operating System (ROS) framework.

**Figure 1:**
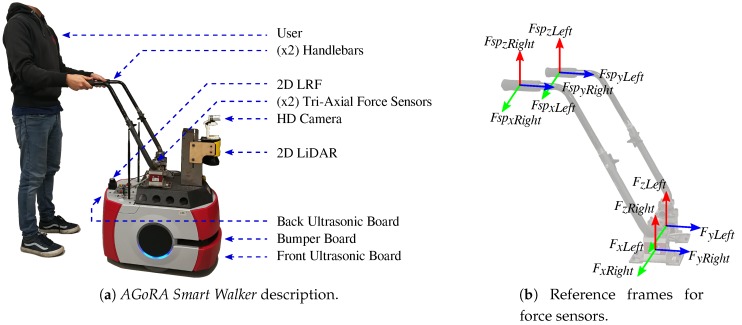
(**a**) The *AGoRA Smart Walker* is a robotic walker mounted on a commercial robotic platform. Several sensor modalities retrofit the walker with user and environment information. (**b**) Coordinate reference frames on handlebars and force sensors.

As shown in Figure 1a, several sensory modalities, actuators, and processing units were implemented and integrated on the *AGoRA Smart Walker*. The *AGoRA Smart Walker* is equipped with: (1) Two motorized wheels and two caster wheels for walker’s propulsion and stability; (2) two encoders and one Inertial Measurement Unit (IMU) to measure walker’s ego-motion; (3) a 2D Light Detection and Ranging Sensor (LiDAR) (S300 Expert, SICK, Waldkirch, Germany) for environment and obstacle sensing; (4) two ultrasonic boards (one in the back and one in the front) for user’s presence detection and low-rise obstacles detection; (5) two tri-axial load cells (MTA400, FUTEK, Irvine, CA, USA) used to estimate the user’s navigation commands; (6) one HD camera (LifeCam Studio, Microsoft, Redmond, WA, USA) to sense people presence in the environment; and (7) a 2D Laser Range-Finder (LRF) (Hokuyo URG-04LX-UG01, Osaka, Japan) for user’s gait parameters estimation.

Additionally, to leverage the *AGoRA Smart Walker*’s processing capabilities, an external computer is used for running several non-critical systems. The communication with the external CPU can be achieved through the walker’s Ethernet and Wi-Fi modules.

As shown in Figure 1b, the position of the force sensors on the platform’s deck is not vertically aligned with the actual supporting points of the user on the handlebars. Essentially, the forces in *y*- and *z*-axis read by the sensors (i.e., $F_{yRight}$, $F_{yLeft}$, $F_{zRight}$ and $F_{zLeft}$) will be a combination of the forces in *y*- and *z*-axis at the supporting points (i.e., $Fsp_{yRight}$, $Fsp_{yLeft}$, $Fsp_{zRight}$ and $Fsp_{zLeft}$). The forces in *x*-axis (i.e, $F_{xRight}$, $F_{xLeft}$, $Fsp_{xRight}$ and $Fsp_{xLeft}$) are discarded, as they do not provide additional relevant information.

### 3.2. Interface Design Criteria

The HREI interface presented in this work takes into account several sensor modalities and control strategies to fulfill several design requirements. The design criteria are grouped in the HRI and REI interfaces that compose the final HREI interface:

- HRI Interface functions:
  − *Recognition of user–walker interaction forces*. The interaction forces between the user and the platform are required to analyze the physical interaction between them.
  − *Estimation of user’s navigation commands*. To provide a shared control strategy, as well as a natural and intuitive HRI, the walker needs to be compliant to the user’s intentions of movement.
  − *Detection of user’s presence and support on the walker*. To ensure safe HRI, the walker movement should only be allowed when the user is properly interacting with it (i.e., partially supporting on the platform and standing behind it).
  − *Estimation of user’s gait parameters*. To adapt the walker’s behavior to each user gait pattern, several gait parameters are computed and analyzed.
  − *Implementation of control strategies*. To provide walker natural response to user’s intentions of movement, it is required to introduce control strategies based on physical HRI between the user and the walker.
- REI Interface functions:
  − *Implementation of a robust navigation system*. To provide a safe and effective REI, the implementation of navigation capabilities is required. Such functions include: map building and edition, autonomous localization and path planning.
  − *Walker motion control*. The execution of desired movements on the walker, relays on a low-level motion control provided by the robotic platform previously described.
  − *Detection of surrounding people*. The navigation system is able to sense obstacles (e.g., people, fixed obstacles and moving obstacles) in the environment as simple physical objects. Therefore, to provide social interaction capabilities between the walker and surrounding people, it is necessary to differentiate among those types of obstacles.
  − *Path adaptation due to social spacing*. To ensure social interaction, the detected surrounding people should modify or adapt the results from the path planning system.
  − *Security restrictions*. A low-level security system is required to ensure safe interaction, even under failure or malfunction of previously described systems.
- Additional functions:
  − *Remote control by therapy supervisor*. The therapy manager should be able to modify the walker parameters, as well as to set the desired control strategy.
  − *Emergency braking system*. To provide an additional safety system, the platform should be equipped with an emergency system based on an external input that completely stops the walker.
  − *Session’s data recording*. The platform should be equipped with a storage system for data recording, in such a way that the information is available for further analysis.

**Figure 2:**
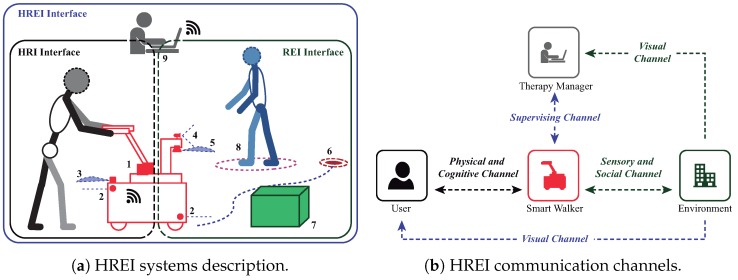
HREI interface model and communication channels. (**a**) HRI and REI systems: (1) Estimation of user interaction forces; (2) low level security rules; (3) laser based estimation of user’s gait parameter; (4) laser-camera fusion scheme for people detection; (5) laser based navigation; (6) motion control for navigation goal reaching; (7) low-rise obstacle avoidance; (8) social spacing for people type obstacles; and (9) therapy supervision. (**b**) Communication channels.

According to the above, Figure 2a illustrates the most relevant systems provided by the HRI and REI interaction interfaces included in our approach.

### 3.3. Interface Communication Channels

Relying on the different interface functions, there are some notable communication channels that provide information exchange between them, as shown in Figure 2b. The communication channels immersed over the HREI interaction are described as follows:

- *User–Walker physical and cognitive channel*. Through this communication channel, the walker’s sensors assess the user’s information (i.e., navigation commands, interaction forces, body weight support and gait parameters). Similarly, the user is able to sense the walker’s behavior through mechanical impedance, safety restrictions, guidance, and response to navigation commands.
- *Walker–Environment sensory and social channel*. The walker’s behavior is also a result of the information retrieved from the environment (e.g., obstacles and the presence people). Such information is used by the walker’s systems to accomplish obstacle avoidance, safety provision, and social interaction.
- *Manager–Walker supervising channel*. A therapy manager is able to remotely assess the session data, as well as override or control walker behavior, if required.
- *Manager–Environment supervising channel*. The environment is also sensed by the natural communication channel with the therapy manager (i.e., visual supervision). Such natural sensing allows the manager to set and control the walker’s behavior.
- *User–Walker–Environment visual channel*. Relying on the visual faculty of the user, the environment and walker behavior is cognitively sensed by the user. This natural communication channel takes place during the HREI loop, however it is not addressed or included in the HREI control strategies.

The following sections describe the systems that compose each interaction interface (i.e., HRI interface and REI interface), as well as the proposed control strategies.

## 4. HRI Interface

Based on the physical interaction between the user’s upper limbs and the walker’s handlebars, the HRI interface is composed of two systems: (A) a gait parameters estimator; and (B) a user’s intention detector.

### 4.1. Gait Parameters Estimator

During gait, the movement of human trunk and center of mass describe oscillatory displacements in the sagittal plane [48]. Thus, in walker assisted gait, the interaction forces between the user and the walker handlebars are associated to the movements of the user’s upper body [44].

In this sense, to implement a proper control strategy based on such interaction forces, a filtering and gait parameter extraction process is required. Consequently, the estimation of the user’s intentions of movement and the user’s navigation commands could be achieved with ease and less likely to be misinterpreted.

According to the above, to carry out filtering processes, a gait cadence estimator (GCE) was implemented. The GCE addresses the gait modeling problem, which is reported in the literature to be solved with several applications of the Kalman filter and adaptive filters [49]. In fact, the Weighted-Fourier Linear Combiner (WFLC) is and adaptive filter for tracking of quasi-periodic signals [49], such as gait related signals (e.g., the interaction force on walker’s handlebars). Therefore, based on the on-line method proposed by Frizera-Neto et al. [50], a GCE was integrated into the HRI interface. This method uses a WFLC to estimate gait cadence from upper body interaction forces.

**Figure 3:**
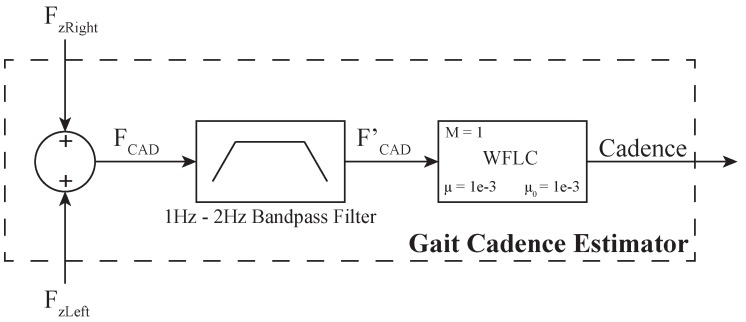
The Gait Cadence Estimator system takes the vertical interaction forces through a filtering process, based on a band-pass filter that eliminates high frequency noise due to walker’s vibrations. Finally, the Weighted-Fourier Linear Combiner filter adaptively estimates the user’s gait cadence.

The two vertical forces (i.e., $F_{zRight}$ and $F_{zLeft}$) are computed to obtain a final force, $F_{CAD}=\left(F_{zRight}+F_{zLeft}\right)/2$. The resulting force, $F_{CAD}$, is firstly passed through a band-pass filter with experimentally obtained cutoff frequencies of 1 Hz and 2 Hz. This filter allows the elimination of signal’s offset and high frequency noise (i.e., mainly due to vibrations between the walker structure and the ground). The filtered force $F_{CAD}^{\prime }$ is fed to the WFLC, in order to estimate the frequency of the first harmonic of $F_{CAD}^{\prime }$. Such frequency represents the gait cadence, which is the final output of the GCE. This process is illustrated in Figure 3.

According to several experimental trials, the users performed significant forces, related to their intentions of movement, along *y*-axis (i.e., $F_{yLeft}$ and $F_{yRight}$, see Figure 1b). It was also observed that the user’s navigation commands were mainly included within the *y*-axis forces. Therefore, the *x-axis* (i.e., $F_{xLeft}$ and $F_{xRight}$, see Figure 1b) forces were discarded. As previously stated, the interaction force signals require a filtering process to remove high frequency noise and signal offset [50]. Thus, a fourth order *Butterworth* low-pass filter was used.

**Figure 4:**
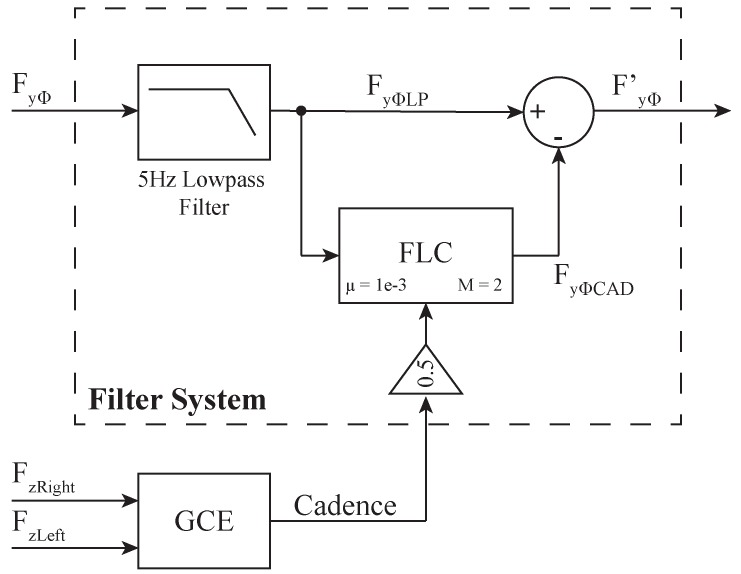
Filter system for *y*-axis forces (Φ means left or right). There is an independent FS for each *y*-axis force (i.e., $F_{yLeft}$ and $F_{yRight}$), composed by a low-pass filter and a FLC filter.

To eliminate gait components from the interaction force signals along *y*-axis, a Fourier Lineal Combiner (FLC) filter in conjunction with the GCE was implemented. Such integration is illustrated in the filtering system (FS) diagram shown in Figure 4. The FS is independently applied to both left and right forces obtaining filtered forces $F_{yLeft}^{\prime }$ and $F_{yRight}^{\prime }$. Thus, Figure 4 denotes $F_{y\Phi }$ as whether $F_{yLeft}$ or $F_{yRight}$ and $F_{y\Phi }^{\prime }$ as whether $F_{yLeft}^{\prime }$ or $F_{yRight}^{\prime }$. The final output $F_{y\Phi }^{\prime }$ of the FS is calculated as the difference between the resulting signal from the low-pass filter (i.e., $F_{y\Phi LP}$) and the output of the FLC (i.e., $F_{y\Phi CAD}$, the cadence signal obtained from each $F_{y\Phi }$ signal).

As shown in Figure 4, the order *M* of the FLC filter was experimentally set to 2, and a 0.5 gain was added between the GCE’s output and the FLC’s frequency input. This gain was set to filter any additional harmonics produced by asymmetrical supporting forces [51]. Moreover, an adaptive gain μ of 0.008 was used.

**Figure 5:**
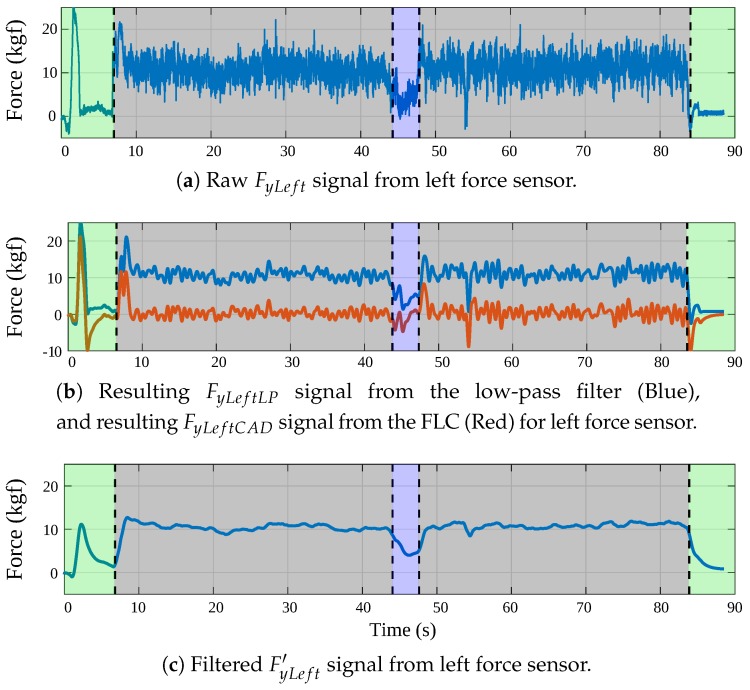
(**a**) Raw $F_{yLeft}$ signal from left force sensor. (**b**) $F_{yLeftLP}$ (Blue), meaning the resulting signal from the low-pass filter, and $F_{yLeftCAD}$ (Red), meaning the resulting signal from the FLC. (**c**) $F_{yLeftLP}$ and $F_{yLeftCAD}$ were subtracted obtaining the filtered signal without gait components, $F_{yLeft}^{\prime }$.

The final linear force *F* and torque τ, applied by the user to the walker, were computed using $F_{yLeft}^{\prime }$ and $F_{yRight}^{\prime }$ (i.e., the *y*-axis forces resulting from the filtering processes) as follows: *F* is computed as the sum of $F_{yLeft}^{\prime }$ and $F_{yRight}^{\prime }$, and τ as the difference between them. For instance, the $F_{yLeft}$ signal obtained from the left force sensor and the implementation of the different filters is presented in Figure 5. The signal obtained corresponds to the readings of the force sensor during a walk along an L-shaped path. Different zones are illustrated in the figure: (1) the green zones show the start and end of the path; (2) the five gray areas denote straight parts of the path; and (3) the blue zone corresponds to the curve to the right, where a reduction of the signal is observed.

### 4.2. User’s Intentions Detector

Starting from the linear force signal and the torque signal, two admittance controllers were implemented to generate walker’s linear velocity and angular velocity responses from user’s intentions of movement. This type of controllers has been reported to provide natural and comfortable interaction in walker assisted gait [28], as they take the interaction forces to generate compliant walker behaviors. Specifically, the implemented admittance controllers emulate dynamic systems providing the user with a sensation of physical interaction during gait assistance. These systems are modeled with two *mass–damper–spring* second-order systems, whose inputs are the resulting force *F* and torque τ (i.e., the force and torque applied to the walker by the user), from the filtered *y*-axis forces. The outputs of these controllers are the linear (*v*) and angular (ω) velocities, meaning the user’s navigation commands.

On the one hand, the transfer function of the linear system is described by Equation (1) (L(s) stands for Linear System), where *m* is the virtual mass of the walker, $b_{l}$ is the damping ratio and $k_{l}$ is the elastic constant. On the other hand, Equation (2) (A(s) stands for Angular System) shows the transfer function for the angular system, where *J* is the virtual moment of inertia of the walker, $b_{a}$ is the damping ratio, and $k_{a}$ is the elastic constant for the angular system. According to this, the static and dynamic behavior, meaning the mechanical impedance of the walker, could be changed by the modification of the controllers parameters.

(1) $$L\left(s\right)=\frac{v(s)}{F(s)}=\frac{\frac{1}{m}}{s^{2}+\frac{b_{l}}{m}s+\frac{k_{l}}{m}}$$

(2) $$A\left(s\right)=\frac{\omega (s)}{\tau (s)}=\frac{\frac{1}{J}}{s^{2}+\frac{b_{a}}{J}s+\frac{k_{a}}{m}}$$

**Figure 6:**
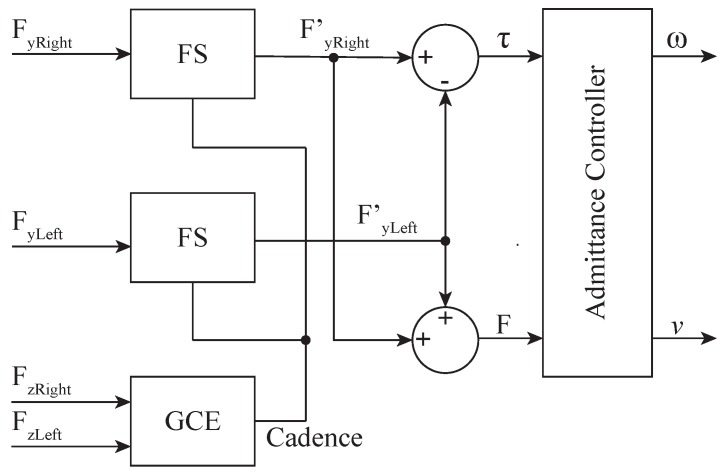
HRI interface system diagram.

Empirically, the authors realized that the values of m=15 Kg, $b_{l}=5$ N·s/m, J=5 Kg·m${}^{2}$ and $b_{a}=4$ N·m·s were appropriate for the purposes of the experimental study. Moreover, $k_{l}$ and $k_{a}$ were used for the walker’s behavior modulation. Figure 6 shows how the two FSs of the GCE and the user’s intention detector are connected.

The next section describes the implemented systems for REI on the walker.

## 5. REI Interface

The REI interface is composed of three main systems: (A) a navigation system; (B) a human detection system; and (C) a low-level safety system.

### 5.1. Navigation System

Navigation during walker-assisted gait is mainly focused on safety provision while guiding the user through different environments. According to the health condition that is being rehabilitated or assisted, the implementation of goal reaching and path following tasks is required. Moreover, such navigation tasks on smart walkers require the consideration of user interaction strategies, obstacle detection and avoidance techniques, as well as social interaction strategies. Particularly, the navigation system presented in this work considers map building, autonomous localization, obstacle avoidance and path following strategies and is based on previous developments of the authors [52].

#### 5.1.1. Map Building and Robot Localization

**Figure 7:**
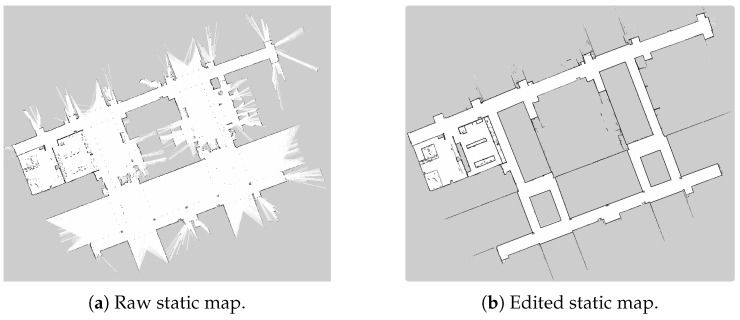
(**a**) Navigation raw static map. (**b**) Navigation edited static map. White means non-obstacle zones, gray means unknown zones and black means obstacles.

Relying on the ROS navigation stack, a 2D map building algorithm, that uses a Simultaneous Localization and Mapping (SLAM) technique to learn a map from the unknown environment was integrated. Specifically, the ROS *GMapping* package for map learning was used [53]. This package is aimed at creating a static map of the complete interaction environment. The static map is made off-line and is focused on defining the main constrains and characteristics of the environment. Figure 7a shows the raw static map obtained at the authors’ research center. This map is also used for the walker on-line localization. For this purpose, the Adaptive Monte Carlo Localization Approach (AMCL) [54] was configured and integrated.

In general, zones such as stairs, elevator entrances, and corridor railings, among others, are defined as non-interaction zones (i.e., mainly due to the risk of collisions). These restrictions are achieved by an off-line editing process of the resulting static map. Further modifications are also required, since LiDARs are light-based sensors and the presence of reflecting objects, such as mirrors, affects their readings. As shown in Figure 7b, the map constitutes a grayscale image, therefore modifications were made by changing colors in the map.

#### 5.1.2. Path Planning and Obstacle Detection

To achieve path planning, 2D *cost-maps* are elaborated from the previous edited map. These *cost-maps* consist of 2D occupancy grids, where every detected obstacle is represented as a cost. These numerical costs represent how close the walker is allowed to approach to the obstacles. Specifically, local and global *cost-maps* are generated. The local *cost-map* is made using readings from the LiDAR that rely on a portion of the edited map, while the global *cost-map* uses the whole edit map. Moreover, these *cost-maps* semantically separate the obstacles in several layers [55]. The navigation system integrated in this work was configured with an *static map layer*, an *obstacle layer*, a *sonar layer* and an *inflation layer* [55]. During the path planning process, the global *cost-map* is used for the restriction of global trajectories. The local *cost-map* restricts the planning of local trajectories, which are affected for variable, moving and sudden obstacles.

The Trajectory Rollout and the Dynamic Window approaches (DWA) were used to plan local paths, based on environment data and sensory readings [56]. As presented in the research of Rösmann et al. [57], this local planner is optimized using a Time Elastic Band (TEB) approach. The information of the environment and global cost-map is used by a global path planner. This planner calculates the shortest collision-free trajectory to a goal point. To do this, the *Dijkstra’s* algorithm was used. Finally, a motion controller takes into account both trajectory plans and generates linear and angular velocity commands to take the walker to each plan’s positions.

**Figure 8:**
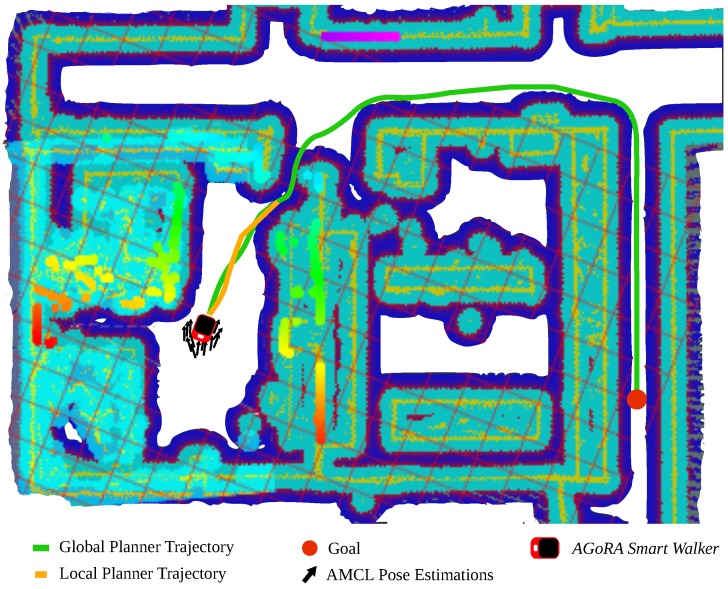
Illustration of a navigation task for the AGoRA Smart Walker reaching a specific goal. Green and orange lines represent local and global trajectories calculated by the path planning system. Light blue and dark blue zones represent the 2D *cost-map* occupancy grid.

Figure 8 shows the trajectories planned by the local and global planner, the positions estimations calculated by the AMCL algorithm, a current goal and the cost-map grid.

### 5.2. People Detection System

**Figure 9:**
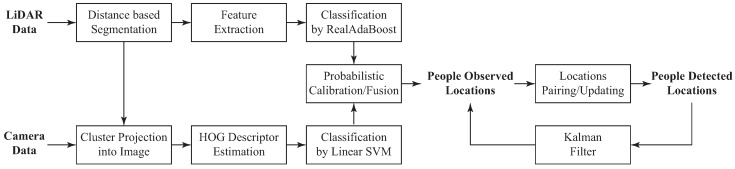
Outline of the people detection system.

The main goal of this module is to complement the performance of the navigation module in the distinction of obstacles regarding to people from simple obstacles (i.e., stationary or mobile objects). This distinction enables the walker with social acceptance and social interaction skills. To achieve this, the people detection system implemented in this work is based on the techniques proposed by Fotiadis et al. [58] and Garzón et al. [59]. Such approaches exploit the localization information provided by the laser of potential humans, in order to reduce the processing time of the camera data. This sensory fusion requires a proper process of calibration. Hence, an extrinsic calibration method was implemented for laser-camera information fusion. Figure 9 illustrates the methodology of the integrated people detection system.

#### 5.2.1. Detection Approach

The people detection system begins with the segmentation of laser data into clusters, based on Euclidean distance differences. These laser clusters are inputs of a process of characteristic extraction [60]. Consequently, these features feed a classification algorithm based on *Real AdaBoost* [61], which is trained off-line with several laser clusters. In parallel, a camera based detection process starts from the projection of each laser cluster into the image frames. As previously mentioned, this projection is accomplished thanks to a calibration process that provides a set of rotation and translation matrices. Such matrices allow the transformation of laser points into the camera frame [62]. From the localization of each cluster, a region of interest (ROI) is defined for the calculation of a Histogram of Oriented Gradients (HOG) descriptor [63]. This HOG descriptor is used by a Linear Support Vector Machine (SVM), which is aimed at classifying the descriptor.

As also proposed in [58], to increase the possibilities to detect a person, the ROI is defined by several adaptive projections, resulting in a group of ROIs in which a person might be.

Both classifiers, *Real AdaBoost* and Linear SVM, are not completely probabilistic methods, since they produce probability distributions that are typically distorted. Such distortions take place as the classifiers outputs constitute signed scores representing a classification decision [64]. To overcome this, a probabilistic calibration method is proposed. The calibration of *Real AdaBoost* scores is achieved by a logistic correction and for the Linear SVM a parametric sigmoid function is used [58]. Afterwards, the outputs of each classifier are passed through an information fusion system, in order to get a unique probabilistic value from both detection methods, resulting in a decision about the presence of people in the environment.

Finally, a tracking process takes into account the previous people observations to generate a final decision about pedestrian locations. As presented by one of the authors, a Kalman filter instance is created for each detection, including those that rely out the image frame [59]. Based on each person’s current and previous position, the filter uses a linear model to calculate people velocities, and consequently achieve the tracking task. A location pairing-updating process is carried out, as presented in [59]. This process is aimed at adding new people locations, updating previous locations, scoring, and removing them.

**Figure 10:**
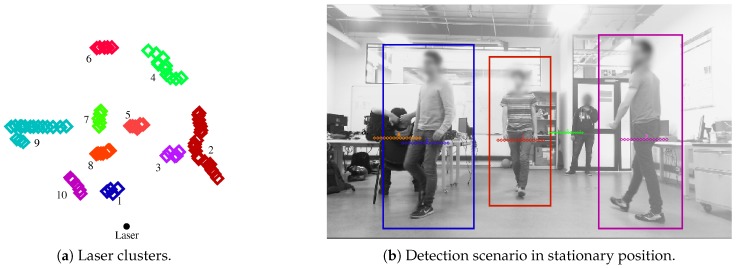
(**a**) Clusters obtained from the segmentation process of laser’s data. (**b**) Three people detected in stationary position.

Figure 10a shows several laser clusters obtained from a LiDAR reading. Figure 10b explains the projection of the clusters into the image, where possible. Likewise, three moving people were detected out four. The laser cluster related to the non-detected person included additional points belonging to walls, therefore its detection was not achieved.

#### 5.2.2. Social Interaction

The navigation system and people detection system are integrated to enable the *AGoRA Smart Walker* with social interaction and social acceptance skills. This is accomplished by adjusting how obstacles are understood by the navigation system. Through the modification of the navigation 2D *cost-map*, these changes are achieved. As described in the navigation system, the obstacles detected in the environment, including people, are represented as equal costs in the 2D *cost-maps*. Therefore, it is necessary to inflate the costs corresponding to a person, in order to avoid the interruption of social interaction zones in the environment. The inflation is made to match the social interaction zone of each person. This is achieved using the information provided by the people detection system, and passing people locations to navigation system. The criteria to inflate the costs are defined by strategies of adaptive spacing in walker–human interactions, as described in [65].

### 5.3. Safety Restrictions System

The *AGoRA Smart Walker* is aimed to be both remotely supervised by a therapy manager, meaning medical staff or technical staff, as well as to be controlled by the user’s intentions of movement. Thus, some security rules were included to constraint the walker’s movement.

#### 5.3.1. User Condition

The walker movement is only allowed if the user is supporting itself on the walker handlebars, as well as standing behind it within an established distance.

#### 5.3.2. Warning Zone Condition

The maximum allowed velocity of the walker is constrained by its distance to surrounding obstacles. A squared shape warning zone is defined in front of the walker, and its dimensions are proportionally defined by the walker’s current velocity. If an obstacle yields within the warning zone, the maximum velocity is constrained.

**Figure 11:**
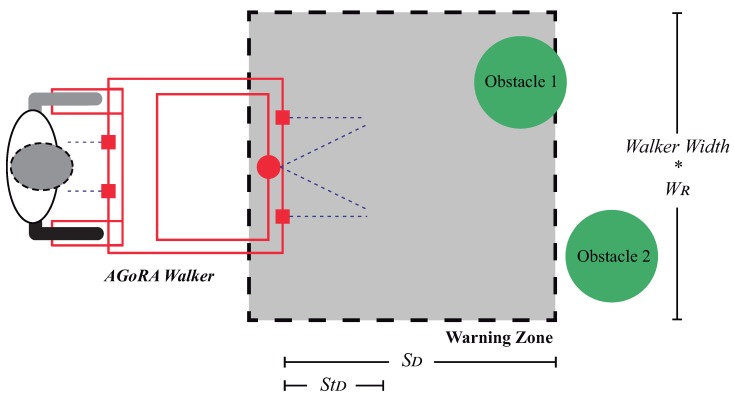
Warning zone shape and parameters for velocity limitation during obstacles presence.

Figure 11 illustrates the warning zone shape and its parameters that change according to the walker’s velocity. The Stop Distance Parameter (STD) determines the minimum distance of the walker to an obstacle before absolute stopping. The Slow Distance Parameter (SD) determines the distance at which obstacles will begin to be taken into account before velocity limitation. Hence, if an obstacle is at distance SD, the walker’s velocity will be slowed. The Width Rate (WR) parameter is the multiplying factor of the warning zone width. When an obstacle is detected within the warning zone, the velocity is limited as described in Equation (3).

(3) $$V_{max}=Slow_{vel}\cdot \frac{D_{obs}-STD}{SD-STD}$$

$D_{obs}$ is the distance to the nearest obstacle and $Slow_{vel}$ is the maximum allowed velocity when an obstacle is the warning zone. Additionally, the $Slow_{vel}$ is continuously adapted by the walker’s velocity, as shown in Table 2. Such values were defined after several experimental trials, in such a way that the warning zone ensures proper stopping of the walker at each velocities range.

## 6. Control Strategies

As previously explained in Section 3, the HREI interface integrates functions from the HRI and REI interfaces, in order to provide efficient, safe and natural interaction. To this end, three control strategies were proposed.

### 6.1. User Control

By the implementation of the HRI interface, the user is able to control the walker’s motion. The gait parameter estimator and the admittance controller are capable of generating velocity commands from the interaction forces. However, the security rules keep ensuring a safe interaction with the environment. Additionally, as the therapy manager is able of controlling the walker’s movement, through a wireless joystick the user’s commands can be revoked or modified.

### 6.2. Navigation System Control

In this control mode, the REI interface has total control of the walker’s movement for providing secure user guidance (i.e., the user’s intentions of movement are ignored). The guidance goals can be whether programmed or on-line modified, while the navigation and social interaction system ensure safety paths. Additionally, the security rules warrant that the walker moves only if the user is supporting and standing in front of the walker.

### 6.3. Shared Control

This strategy combines the navigation velocity commands and the user’s intentions of movement for walker’s control granting. The user’s intentions are calculated using F and τ, as a vector of magnitude equals to the normalized F, with proportional orientation to the exerted τ. Equation (4) illustrates the calculation of intention vector’s orientation, where $Max_{angle}$ is the maximum turn angle allowed and MET is the maximum exerted torque.

(4) $$\theta {\left(t\right)}_{usr}=Max_{angle}\cdot \frac{\tau (t)}{MET}$$

**Figure 12:**
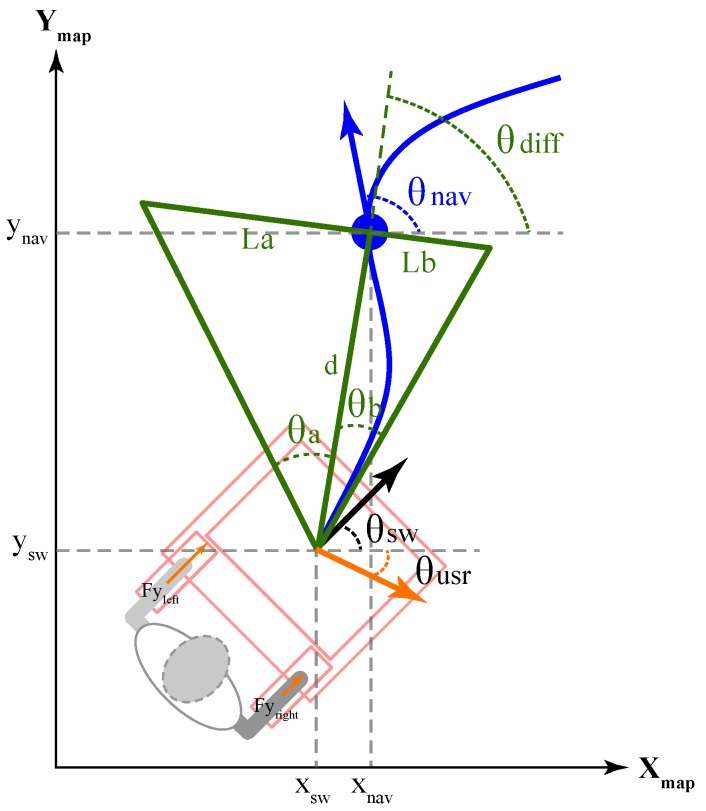
Estimation of possible user’s intentions area.

To estimate the control granting (i.e., walker control by the user or by the navigation system), the user’s intentions are compared with the navigation path, to obtain the final pose to be followed by the walker. Specifically, as shown in Figure 12, for the nearest path point ($x_{nav}$, $y_{nav}$) to the current walker position at ($x_{sw}$, $y_{sw}$), a range of possible user intentions is calculated (i.e., the range where the control is granted to the user). The positions are calculated in the map coordinate reference frame, since the navigation system generates the path plans in such reference frame.

In Figure 12, the range of possible intentions is calculated as a triangle-shaped window, which is formed by: (1) $\theta_{sw}$, the current orientation of the walker; (2) $\theta_{usr}$, the current user’s intention of movement; (3) $\theta_{nav}$, the orientation of the next and nearest path point; and (4) *d*, the Euclidean distance from the walker position to the next pose. The geometric parameters for the window formation are described in Equations (5)–(8). A window scaling factor $Wind_{w}idth$ is used to adapt the window area. Graphically, the window is formed by two right-angled triangles. These smaller triangles are constituted with height *d*, bases La and Lb, and auxiliary angles $\theta_{a}$ and $\theta_{b}$.

(5) $$L_{a}=\frac{Win_{width}\cdot \left(\theta_{nav}-\theta_{sw}\right)}{Max_{angle}}$$

(6) $$L_{b}=Win_{width}-L_{a}$$

(7) $$\theta_{a}=tan^{-1}\left(\frac{L_{a}}{d}\right)$$

(8) $$\theta_{b}=tan^{-1}\left(\frac{L_{b}}{d}\right)$$

If the user’s intention of movement lies in the described window, the control is granted to the user. Otherwise, if the user’s objective lies outside the area of possible movements, a new path pose is computed. This new pose is calculated to be within the area of possible movements. To this end, both $x_{nav}$ and $y_{nav}$ define the new pose position and the new pose orientation ($\theta_{nxt}$) is defined as presented in Equation (9):

(9) $$\theta_{nxt}=\left\{\begin{array}{ccc} \theta_{nav}, & if & \theta_{diff}-\theta_{a}\leq \theta_{usr}\leq \theta_{diff}+\theta_{b}\\ \theta_{diff}-\theta_{a}, & if & \theta_{usr}<\theta_{diff}-\theta_{a}\\ \theta_{diff}+\theta_{b}, & if & other \end{array}\right.$$

where $\theta_{diff}$ is estimated as shown in Equation (10) and represents the relative center of the window of possible movements.

(10) $$\theta_{diff}=sin^{-1}\left(\frac{y_{nav}-y_{sw}}{d}\right)$$

## 7. Experimental Tests

To evaluate the described HREI interface, several performance and usability tests were proposed, regarding the control strategies previously described. The main goal of these tests was to assess the performance of every module of the *AGoRA Smart Walker*, both independently and simultaneously. Several healthy subjects were recruited to voluntarily participate in the validation study. Specifically, seven volunteers conformed the validation group (6 males, 1 female, 33.71±16.63 y.o., 1.69±0.056 m, 65.42±7.53 kg) with no gait assistance requirements accomplished the tests that are further presented (see Table 3 for additional information).

The experimental trials took place at the laboratories building of the Colombian School of Engineering. A total of 21 trials divided into 7 sessions were performed. Every session consisted in three different trials of each specific control mode (i.e., user control, navigation system control and shared control). At the beginning of each session, the order in which the modes of operation were going to be evaluated was randomized. Likewise, before each trial the volunteers were instructed in the behavior of control mode, allowing them to interact with the platform. During trials, the researchers stayed out of the session environment to avoid interfering with the tasks achievement. At the end of each trial, a data log including user and walker’s information was stored for further analysis purposes.

According to the above, the obtained results under each control mode are presented in the following sub-sections.

### 7.1. User Control Tests

**Figure 13:**
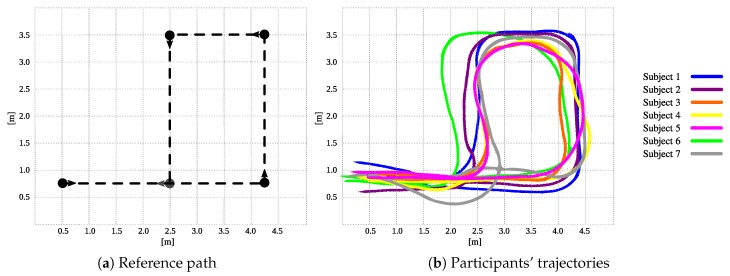
(**a**) Reference path for user control tests based on a square-shaped trajectory. Landmarks and path direction were indicated through reference points at path corners. (**b**) Trajectories achieved by the nine participants under user control trials.

**Figure 14:**
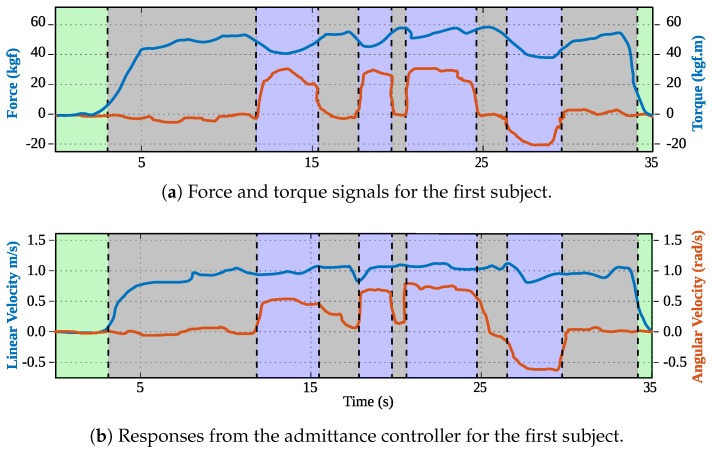
(**a**) Force (blue) and torque (orange) signals during the trajectory for the first subject. (**b**) Linear (blue) and angular (orange) velocities obtained from the admittance controller during the trajectory for the first subject.

The volunteers were asked to achieve a square-shaped trajectory by following several landmarks. Figure 13a illustrates the reference trajectory to be followed by the participants and Figure 13b illustrates the achieved trajectories by the participants. Under this control mode, the only active systems were those corresponding to the HRI interface. The trajectory was aimed at assessing the capabilities of the interface to respond to the users’ intentions of movement and adapt to their gait pattern. Specifically, the gait parameter estimator was responsible for acquiring and filtering the force and torque signals due to the physical interaction between the walker and the user. As an explanatory result, Figure 14a shows the filtered signals regarding to force and torque for subject 1. The user’s intentions detector was in charge of generating the linear and angular speed control signals of the walker. Figure 14b shows the speed signals for subject 1. Similarly, the low level security system was running in parallel, in such a way that collisions were avoided. Specifically, no collisions took place during these trials.

During the execution user control trials, higher differences were encountered between the ideal and the achieved paths at the trajectory corners. Accordingly, the 90-degree turns were more difficult to accomplish by the participants, as the *AGoRA Walker* axis of rotation is not aligned with the user’s axis of rotation. However, such kind of turns should be avoided as they risk user’s stability and balance. Thus, less steep turns are more natural and safer for the users.

### 7.2. Navigation System Control Tests

To evaluate the path following and security restrictions capabilities alongside the people detection system, a preliminary guidance trial with one subject was performed in presence of people. The volunteer user was guided through a random path previously programmed, while overcoming both regular and people obstacles in the environment. Additionally, the navigation system was configured with: (1) minimum turning radius of 15 cm, to avoid steeped curves planning; (2) local planner frequency of 25 Hz; (3) global planner frequency of 5 Hz; and (4) maximum linear velocity of 0.3 m/s and maximum angular velocity of 0.2 rad/s.

**Figure 15:**
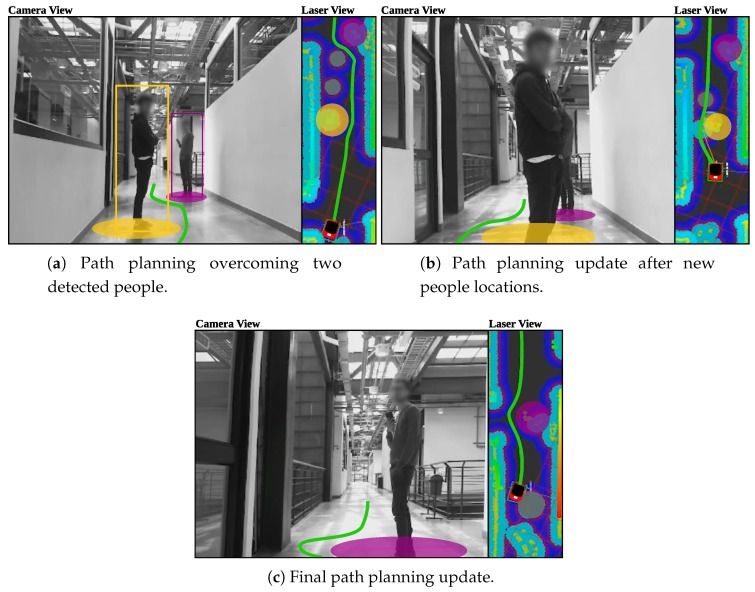
Navigation and people detection systems during guidance task. Yellow and purple squares represent people obstacles detected by both camera and laser. Yellow and purple circles represent people obstacles only detected by the laser, as well as the obstacles costs inflations. Gray circles show old obstacles that will be removed once the walker senses such areas again. Green line illustrates the path.

Figure 15 illustrates the carried out test in three different states. The first state shows the planned trajectory according to the initial environment sense, as shown in Figure 15a. The second state in Figure 15b presents an update in the trajectory due to new people locations. Although the most proximate person to the walker is not detected by the camera, laser readings allows the person’s position tracking and therefore its detection. Finally, Figure 15c illustrates the avoiding of another person, while continuing with the guidance task.

**Figure 16:**
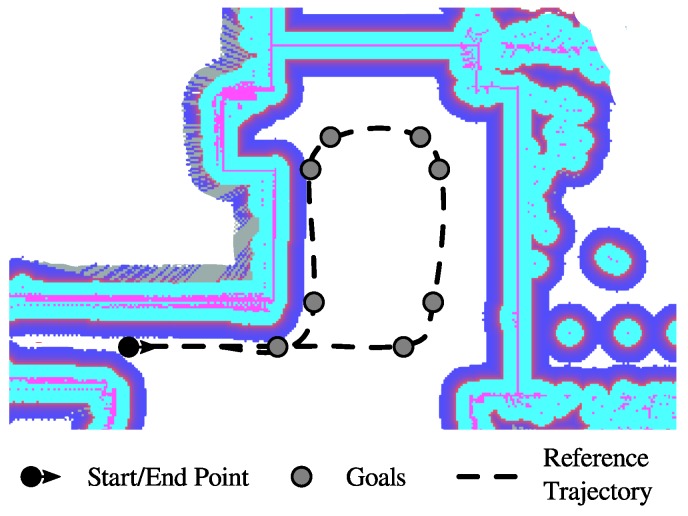
Reference trajectory and goals for the guiding task.

In addition to the above, the guiding capability of the navigation system was also validated on the seven volunteers who participated in the study. Specifically, the predefined path goals presented in Figure 16 were configured in the navigation system to form a desired trajectory. The reference trajectory was designed to be similar to the reference path used for the user control trials. However, the trajectory corners were designed as soft turn curves, in such a way that the user’s balance and stability were not compromised. During the seven trials, no significant differences were encountered in the achieved trajectories, no collisions took place and the mean guidance task time was 53.06±2.15 s. The participants were asked to perceive their interactions with the *AGoRA Walker* during the guiding task.

### 7.3. Shared Control Tests

To assess the shared control performance, each volunteer was asked to follow the reference trajectory previously presented in Figure 16. Under this control mode, the participants were partially guided by the navigation system. Likewise, before each trial the volunteers were informed that their intentions of movement would be taken into account. The Table 4 summarizes main findings for each trial.

The results presented in Table 4 suggest proper capabilities of the shared control strategy to effectively guide the participants through a specific trajectory. Six subjects achieved the full reference path by reaching its ten intermediate goals. Specifically, one subject did not complete the task by only reaching eight goals. This result is due to a random false obstacle perceived at the ninth goal, resulting in the blocking of the path planning module. Regarding the task completion times, the mean task time obtained for all the participants was 67.55±11.25 s. The differences among these times is mainly supported by the fact that the linear speed was totally controlled by the user intentions of movement. Accordingly, the obtained mean linear speed was 0.33±0.07 m/s. Finally, to evaluate the control granting behavior under this mode, the percentage of user control was estimated. This ratio was calculated taking into account the total time of user control and the overall task time. A mean percentage of 66.71±6.26% was obtained. The user control occurred mainly in the straight segments of the trajectory, since at the trajectory curves the users’ intentions of movement did not completely matched to the planned path.

### 7.4. Questionnaires Responses

To qualitatively assess the interactions between the participants and the *AGoRA Walker*, at the end of each trial, the volunteers were asked to fill out a usability questionnaire to obtain instant perceptions of the mode of operation. The participants were also encouraged to highlight perceptions regarding the interaction with the smart walker. Regarding the perception questionnaire, based on the UTAUT models in [66,67], an acceptance and usability questionnaire was designed. The questionnaire was adapted to be relevant to the interaction with the AGoRA Walker (see Table 5 for further details).

**Figure 17:**
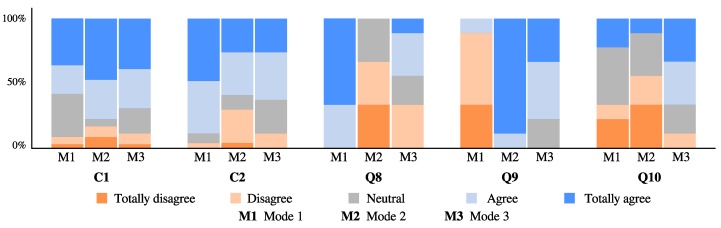
Acceptance and usability questionnaire results: Mode 1, user control; Mode 2, navigation system control; Mode 3, shared control.

The Likert data obtained from the acceptance and usability questionnaires were aimed at assessing the participants’ perceptions of the interaction with the *AGoRA Walker*. For analysis purposes, the answers from Questions Q1–Q4 were grouped into a single category (C1), since they evaluated the attitude towards the device and the expected performance. Similarly, the answers from Questions Q5–Q7 were grouped into another category (C2), as they evaluated the perceived effort and anxiety of the interaction with the device. Finally, Questions Q8–Q10 were aimed at assessing the behavior perception of each control mode. However, the answers from these question were independently analyzed, in order to find differences between them. The questionnaire responses are presented in Figure 17, illustrating the percentage of opinions in each category (i.e., C1 and C2), as well as in Questions Q8–Q10 for each Likert item.

Relying on the questionnaire responses for Categories C1 and C2, a direct measure of the interaction perception in the experimental sessions can be obtained. Consequently, resembling survey answers were obtained under each control mode with major positive distributions. These results might suggest safe, natural and intuitive interactions perceived by the volunteers who participated in the study. Moreover, some participants stated additional comments regarding to the navigation control mode. Specifically, the volunteers suggested that at specific trajectory points the device stopped, in such a way that the path following task was not very comfortable. These impressions occurred at several trajectory goals, since the navigation system was configured to reach them at specific orientations.

To analyze the participants’ behavior perception under each control mode, the responses from Questions Q8–Q10 were statistically analyzed. As found in [68,69], Mann–Whitney–Wilcoxon (MWW) tests have shown optimal results comparing Likert data for small sample sizes MWW. Therefore, the MWW test was used to assess differences in the perception of each control mode. Specifically, Table 6 summarizes the *p* values obtained for each paired test between control modes (i.e., Mode 1, user control; Mode 2, navigation system control; and Mode 3, shared control).

As can be seen in Table 6 and Figure 17, significant differences were encountered among all participants responses for Question Q8. Such outcome may suggest that all participants perceived the ability of the interface to respond to their intentions of movement. Likewise, responses for question Q9 showed significant differences between two paired tests (i.e., Mode 1 vs. Mode 2 and Mode 1 vs. Mode 3), indicating that participants perceived modifications in the walker behavior. Finally, regarding Question Q10, a significant difference was only obtained for paired test between Mode 2 and Mode 3. Such behavior might be supported by the fact that both navigation system control and user control work together under the shared control mode.

## 8. Conclusions and Future Work

An HREI interface, composed by HRI and REI interfaces, was developed and implemented on a robotic platform for walker assisted gait. The robotic platform was equipped with two handlebars for forearm support and several sensory modalities, in order to emulate the performance and capabilities of an SW. Within the HREI interface design criteria, the following functions are found: estimation of user’s intentions of movement, providing of a safe and natural HRI interaction, implementation of a navigation system alongside a people detection system for social interaction purposes, and the integration of a set of control strategies for intuitive and natural interaction.

To validate the platform performance and interaction capabilities, several preliminary tests were conducted with seven volunteer users with no gait requirements reported. Specifically, data were collected from 21 trials divided into seven sessions, where all participant interacted with each control mode. Regarding the user control mode, a squared-shaped trajectory was proposed to be followed by each participant. The achieved trajectories for all the volunteers, as well as the admittance responses for a specific subject were presented. According to the participants’ performance under this control mode, preferences for less steeped curves were found. Concretely, the participants did not strictly execute 90-degree turns at trajectory corners. Such behavior is mainly supported by the not aligned axes of rotation of the walker and the users. Moreover, ignoring path corners allowed the participants to ensure balance and stability during walking.

The validation trials were also aimed at assessing the performance of the navigation system in guidance tasks, as well as at evaluating the performance of the navigation and people detection systems working together. Specifically, an isolated preliminary test with a volunteer was carried out to evaluate the capabilities of the platform for overcoming environments with people, even when sudden changes in obstacles locations. In the preliminary test, both navigation and people detection systems were executed at a maximum frequency of 4 Hz, due the on-board computational limitations. To ensure user’s balance and stability, the trajectory planning was configured to prefer curves with minimum turning radius of 15 cm. Although collisions and system clogging were not presented, the implementation of the REI on clinical or crowded scenarios should required higher computational resources. Regarding the validation trials with the seven volunteer users, a reference trajectory composed by 10 intermediate goals was proposed. All participants experienced the navigation system control completely achieving the reference path with no collisions.

Regarding the assessment of the shared control mode, a path following task was also proposed. Under this control mode, the participant’s intentions of movements and the navigation system cooperatively controlled the platform. Specifically, the linear speed was totally controlled by the users. Similarly, the angular speed was controlled according to the shared control strategy estimations. To ensure participant’s balance and stability, minimal turning radius of 15 cm were also configured. Among the participants trials, a mean percentage of user control of 66.71±6.26 was obtained. Concretely, the control of the platform was mainly granted to the user at straight segments of the trajectory, since the participants’ did not have exact information about the reference trajectory. According to the geometrical model implemented for the shared control strategy, more strict or more flexible behaviors can be configured by modifying the dimensions of the interaction window. Such modifications can potentially be implemented in rehabilitation scenarios in order to provide different levels of assistance. Specifically, early stages of physical and cognitive rehabilitation processes might benefit from more rigorous interaction windows, ensuring a higher percentage of control of the navigation system.

A qualitative assessment of the platform performance and interaction capabilities relying on an acceptance and usability questionnaire was carried out. The participants’ attitude towards the device, as well as the performance and behavior perception were evaluated. According to the survey responses, the participants perceived a mostly positive interaction with the platform. Specifically, the questionnaires showed natural, safe and intuitive interactions under all the control modes. Regarding the behavior perception, significant differences were statistically found between the control modes. Slightly negative distributions were found for the navigation system control for C2 questions. These questions were aimed at evaluating effort and anxiety perceptions, which where experience by some participants. Particularly, two volunteers stated that the navigation system suddenly stopped at specific points of the trajectory. Such behavior was mainly due to the system configuration to reach goals at specific orientations.

Future works will address extensive evaluations of social interactions between the walker and people in the environment, by implementing several avoidance strategies, as well as algorithms for recognition of social groups interactions. Similarly, the assessment of the interface here proposed in clinical and rehabilitation scenarios will be achieved. Specifically, validation studies will firstly be carried out on post-stroke patients as they require a lower assistance level than SCI and CP patients. These validation studies will be aimed at analyzing specific relationships between the users’ characteristics and the interaction performance. Moreover, according to the the *AGoRA Walker*’s handlebars configuration, the platform might be classified as an assistance SW. Therefore, the HREI interface will be implemented and validated on a rehabilitation SW. Additional developments will seek to implement feedback strategies for the user under each control mode, in order to pursue better performance and interaction perceptions. Future works will also address the implementation of the presented interface on an SW that cooperates with an exoskeleton for gait assistance and rehabilitation. Finally, the integration of a cloud based system could leverage processing capabilities, resulting in better performance results.

## Figures and Tables

**Table 1:** Related works involving smart walkers with the integration of interfaces for Human–Robot–Environment Interaction.

Walker	Type	Sensory Interface	Internal Modules	Modes of Operation	Shared Control Strategies	Social Interaction
GUIDO [22]	Active	- Force sensors - LRF - Sonars - Encoders	- Autonomous navigation - Detection of user’s intentions - Sound feedback	- Supervised - Autonomous	-	-
XR4000 [23]	Active	- Force sensors - LRF - Sonars - Infrared sensors - Encoders	- Autonomous navigation - Detection of user’s intentions	- Free - Supervised - Autonomous	Shared walker steering on active mode	
ASBGo++ [21,24,25]	Active	- Force sensors - LRF - Sonar - Infrared sensors - Camera - Encoders	- Autonomous navigation - Detection of user’s intentions - Gait monitoring - User position feedback	- Free - Supervised - Autonomous	-	-
JARoW [26,27]	Active	- Infrared sensors - Encoders - LRFs	- User position estimation and prediction - Obstacle avoidance	- Free - Supervised	-	-
NeoASAS [14]	Active	- Force sensors	- Detection of user’s intentions	- Free	-	-
UFES [16,28]	Active	- Force sensors - LRF - IMUs - Encoders	- Path following - Obstacle avoidance - Detection of user’s intentions - Gait monitoring	- Free - Supervised - Feedback	Spatially modulated admittance control, visual feedback	-
PAMM [29]	Active	- Force sensors - Sonars - Camera - Encoders	- Autonomous navigation - Health monitoring	User control, path following control	Adaptive and shared admittance controller	-
MOBOT [17,30,31,32]	Active	- Force sensors - LRFs - Cameras - Kinect sensors - Microphones	- Autonomous navigation - Detection of user’s intentions - Speech and gesture recognition - Body pose estimation - Gait Analyzer	Walking assitance, sit-to-stand assistance, nurse type	Adaptive control based on context	-
CAIROW [33]	Active	- Force sensors - LRFs	- Environment analyzer - Force analyzer - Gait analyzer	Context aware mode	Adaptive system parameters	-
ISR-AIWALKER [34,35]	Active	- Force sensors - Kinect sensor -Encoders - Leap motion sensor - RGB-D Camera	- Detection of user’s intention - Gripping recognition - Gait analyzer - Autonomous navigation	- Supervised - Navigation aided	Aided user intent by navigation system	-
COOL Aide [36]	Passive	- Force sensors - LRF - Encoders	- Autonomous navigation - Detection of user’s intentions	- Supervised	Shared control based on obstacles and user’s intentions	-
Wachaja et al. [37]	Passive	- LRF - Tilting LRF	- 3D Mapping and localization - Obstacle avoidance - Vibrotactile feedback	- Single feedback - Multiple feedback	-	-
MARC [38,39]	Passive	- Sonars - Infrared sensors - Encoders	- Path following - Obstacle avoidance	Warning mode, safety braking mode and braking and steering mode	Shared walker steering	-
c-Walker [40]	Passive	- Kinect like sensor - RFID reader - IMU - Camera - Encoders	- Autonomous navigation - People detection and tracking - Guidance	Acoustic feedback, mechanic feedback and haptic feedback	Shared walker steering	Social Force Model for path planning

**Table 2:** Warning zone parameters adaption.

Walker’s Velocity (ms)	Warning Zone Parameters		
	STD (m)	SD (m)	WR
≤0.3	0.3	0.6	1.0
≤0.4	0.3	0.8	1.2
≤0.5	0.3	1.0	1.4
≤0.6	0.3	1.2	1.5
≤0.8	0.3	1.4	2.0
>0.8	0.3	2.0	3.0

**Table 3:** Summary of volunteers who participated in the study.

Subject	Age (y.o.)	Height (m)	Weight (kg)	Gender
1	23	1.76	65	Male
2	23	1.77	72	Male
3	23	1.65	62	Female
4	61	1.67	65	Male
5	23	1.72	69	Male
6	59	1.60	50	Male
7	24	1.70	75	Male

**Table 4:** Summary of the results obtained for shared control trials.

Subject	Achieved Goals	Task Time (s)	Mean Linear Speed (m/s)	Percentage of User Control (%)
1	10	63.94	0.34	69.19
2	10	71.46	0.34	71.63
3	10	48.38	0.46	53.66
4	10	83.45	0.23	62.55
5	10	64.54	0.34	68.25
6	8	80.8	0.21	73.99
7	10	60.29	0.37	67.71

**Table 5:** Acceptance and usability questionnaire used in the study.

No.	Question
Q1	I think the robotic device makes me feel safe
Q2	I think the robotic device was easy to use
Q3	I think most people would learn to use this device quickly, it is intuitive
Q4	I think the device guides me well
Q5	I think my experience interacting with the device was natural
Q6	I think my experience interacting with the device was intuitive
Q7	I think my experience interacting with the device was stressful.
Q8	In this session, I felt that I had control of the device
Q9	In this session, I felt that the device had the control of the path to be followed
Q10	In this session, I felt that the device control was shared with me

**Table 6:** Mann–Whitney–Wilcoxon *p* values for paired tests among Q8, Q9 and Q10. *p* values in bold illustrate significant differences encountered, meaning p≤0.05.

Question	Mode 1 vs. Mode 2	Mode 1 vs. Mode 3	Mode 2 vs. Mode 3
Q8	**0.02**	**0.02**	**0.05**
Q9	**0.02**	**0.02**	0.08
Q10	0.37	0.136	**0.04**

## Abbreviations

The following abbreviations are used in this manuscript:

SCI	Spinal Cord Injury
CP	Cerebral Palsy
WHO	World Health Organization
SW	Smart Walker
SWs	Smart Walkers
HRI	Human–Robot Interaction
HREI	Human–Robot–Environment Interaction
REI	Robot–Environment Interaction
COOL Aide	CO-Operative Locomotion Aide
ROS	Robotic Operating System
IMU	Inertial Measurement Unit
LiDAR	Light Detection and Ranging Sensor
HD	High Definition
LRF	Laser Range Finder
CPU	Central Processing Unit
$F_{xRight}$	Force along the *x*-axis on the right load cell
$F_{xLeft}$	Force along the *x*-axis on the left load cell
$F_{yRight}$	Force along the *y*-axis on the right load cell
$F_{yLeft}$	Force along the *y*-axis on the left load cell
$F_{zRight}$	Force along the *z*-axis on the right load cell
$F_{zLeft}$	Force along the *z*-axis on the left load cell
$Fsp_{xRight}$	Force along the *x*-axis on the right supporting point
$Fsp_{xLeft}$	Force along the *x*-axis on the left supporting point
$Fsp_{yRight}$	Force along the *y*-axis on the right supporting point
$Fsp_{yLeft}$	Force along the *y*-axis on the left supporting point
$Fsp_{zRight}$	Force along the *z*-axis on the right supporting point
$Fsp_{zLeft}$	Force along the *z*-axis on the left supporting point
GCE	Gait Cadence Estimator
WFLC	Weighted-Fourier Linear Combiner
$F_{CAD}$	Resulting force used to estimate user’s gait cadence
$F_{CAD}^{\prime }$	Filtered $F_{CAD}$ force
FLC	Fourier Lineal Combiner
FS	Filtering System of forces along *y*-axis
$F_{y\Phi }$	Representation of whether $F_{yLeft}$ or $F_{yRight}$
$F_{yLeft}^{\prime }$	Filtered $F_{yLeft}$
$F_{yRight}^{\prime }$	Filtered $F_{yRight}$
$F_{y\Phi }^{\prime }$	Representation of whether $F_{yLeft}^{\prime }$ or $F_{yRight}^{\prime }$
$F_{y\Phi LP}$	Resulting $F_{y\Phi }$ signals after low-pass filter
$F_{y\Phi CAD}$	Cadence signals obtained from the FLC
*M*	Order of the FLC filter
μ	Adaptive gain of the FLC filter
*F*	Final force applied to the walker by the user
τ	Final torque applied to the walker by the user
$F_{yLeftLP}$	Resulting signal from the low-pass filter for $F_{yLeft}$
$F_{yLeftCAD}$	Resulting signal from the FLC for $F_{yLeft}$
*v*	Linear velocity generated with an admittance controller
ω	Angular velocity generated with an admittance controller
L(s)	Second order system for linear velocities generation
*m*	Virtual mass of the walker
$b_{l}$	Damping ratio for L(s)
$k_{l}$	Elastic constant for L(s)
A(s)	Second order system for angular velocities generation
*J*	Virtual moment of inertia of the walker
$b_{a}$	Damping ratio for A(s)
$k_{a}$	Elastic constant for A(s)
SLAM	Simultaneous Localization and Mapping
AMCL	Adaptive Monte Carlo Localization Approach
TEB	Time Elastic Band
ROI	Region of interest in the camera image
HOG	Histogram of Oriented Gradients
SVM	Support Vector Machine
STD	Stop Distance Parameter
SD	Slow Distance Parameter
WR	Width Rate
MET	Maximum Exerted Torque
$x_{n}av$	X position of nearest path point
$y_{n}av$	Y position of nearest path point
$x_{s}w$	X position of the walker
$y_{n}av$	Y position of the walker
$\theta_{sw}$	Orientation of the walker
$\theta_{usr}$	Orientation of user’s intention of movement
$\theta_{nav}$	Orientation of nearest path point
*d*	Euclidean distance from the walker position to the next pose
La	Base of first right-angled triangle
Lb	Base of second right-angled triangle
$\theta_{a}$	Auxiliary angle for first right-angled triangle
$\theta_{b}$	Auxiliary angle for first right-angled triangle
$Win_{width}$	Scaling factor of triangle-shaped window
$\theta_{nxt}$	Orientation of next path pose
$\theta_{diff}$	Relative orientation of the triangle-shaped window center
